# A taxonomic summary of *Aphelidiaceae*

**DOI:** 10.1186/s43008-019-0005-7

**Published:** 2019-06-07

**Authors:** Peter M. Letcher, Martha J. Powell

**Affiliations:** Department of Biological Sciences, The University of Alabama, 1332 SEC, Box 870344, 300 Hackberry Lane, Tuscaloosa, AL 35487 USA

**Keywords:** *Aphelidiomycota*, *Aphelidium*, Basal groups, *Paraphelidium*, *Amoeboaphelidium*, *Pseudaphelidium*

## Abstract

Aphelids are parasitoids of various algae and diatoms, and in a recent classification are contained in family *Aphelidiaceae*, phylum *Aphelidiomycota*, kingdom *Fungi*. Family *Aphelidiaceae* (the only family in the phylum) is composed of four genera: *Aphelidium*, *Paraphelidium*, *Amoeboaphelidium*, and *Pseudaphelidium*. All species are known morphologically, and most have been illustrated. Few have been examined ultrastructurally, and even fewer have been sequenced for molecular comparisons. Recent studies in molecular phylogenetics have revealed an abundance of related environmental sequences that indicate unrealized biodiversity within the group. Herein, we briefly summarize the history of aphelids and acknowledge the controversy of placement of the group with related organisms. With light microscopic images and transmission electron micrographs, we illustrate typical life cycle stages for aphelids, provide updated descriptions and taxonomy for all described species, and provide a key to the species.

## INTRODUCTION

Aphelids (*Aphelidiaceae*, *Aphelidiomycota*) are a group of obligate endoparasitoids of various common algae and diatoms. We employ the term “parasitoid” for these organisms, as eventually the infected host cell is consumed and killed, although in multicellular hosts, uninfected cells adjacent to those with infection remain viable. The type, *Aphelidium deformans*, was described more than 130 years ago (Zopf 1885). Among the four described genera, *Aphelidium*, *Amoeboaphelidium*, and *Paraphelidium* occur in freshwater habitats, while *Pseudaphelidium* is found in marine environments (Karpov et al. 2017a; Scherffel 1925; Schweikert and Schnepf 1996; Zopf 1885). Currently, *Aphelidium* is composed of seven species, *Amoeboaphelidium* of five, *Paraphelidium* of two, and *Pseudaphelidium* is monotypic. Thallus morphology has been illustrated for all taxa except *Am. achnanthis*, for which only a written description exists. A minority of taxa have been examined for their zoospore and thallus ultrastructure. Although even fewer have been sequenced for molecular comparisons, recent advances in molecular phylogenetics have revealed an abundance of related environmental sequences that indicate hitherto unrealized biodiversity within the group (e.g. Karpov et al. 2014a, 2017a).

In a recent high-level classification (Tedersoo et al. 2018), aphelids are placed as an early-diverging lineage in kingdom *Fungi*, and we adhere to this classification here. Although aphelids are considered opisthokonts because of their posteriorly uniflagellate zoospores, the classification of aphelids as *Fungi* (Tedersoo et al. 2018) is not without controversy. Gromov (2000) and Karpov et al. (2013) thoroughly discussed historical interpretations of the phylogenetic affinities of aphelids, the organisms originally having been considered extremely divergent “fungal animals”– organisms demonstrating a fungal-like life-cycle, but having an amoeboid trophic stage. Later, aphelids were for a time considered protists (class *Rhizopoda*, order *Proteomyxida*) (e.g. Hall 1953). With molecular phylogeny analyses, Karpov et al. (2013) showed that *Aphelidea* was sister to both *Microsporidia* and *Cryptomycota*, and all three phyla form a separate monophyletic lineage sister to traditional fungi, which include *Dikarya* (*Ascomycota* and *Basidiomycota*), paraphyletic *Zygomycota*, and *Chytridiomycota*. Karpov et al. (2013) erected a superphylum *Opisthosporidia* “… named by word combination of Opisthokont and sporae, making reference to the specialized penetration apparatus of the spore (in *Microsporidia*) and cyst (in the two other phyla) characteristic for all three phyla *Microsporidia*, *Cryptomycota*, and *Aphelida*” (Karpov et al. 2013). *Opisthosporidia* is sister to the traditional fungi. Most recently, Torruella et al. (2018), analyzing various protein datasets in multi-gene phylogenomic analyses, place aphelids as the closest relatives of *Fungi* to the exclusion of *Cryptomycota* and *Microsporidia*, suggesting that *Fungi* evolved from an aphelid-like ancestor that lost phagotrophy and became osmotrophic. Nonetheless, a clear and convincing taxonomic repository for the aphelids remains to be determined. Alternatively, Adl et al. (2019), in a classification of eukaryotes that adopted a hierarchal system without formal rank designations, retained *Aphelidea* in the *Opisthosporidia* (*Fungi*), but noted “… the placement of *Aphelidea* in *Opisthosporidia* is unstable and may change”.

**Fig. 1:**
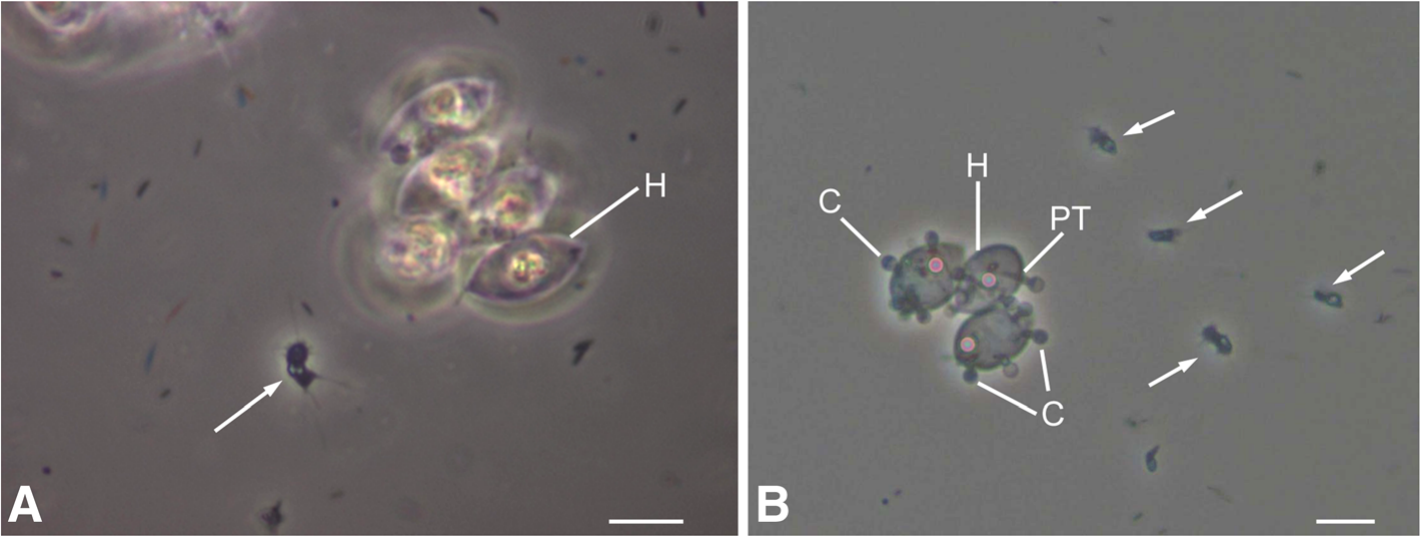
Light microscopic images of *Aphelidium protococcorum* (FD 95), representative of *Aphelidiaceae*. **a** Motile zoospore (arrow) in vicinity of uninfected host (H) (*Scenedesmus*) cells. **b** Motile zoospores (arrows) and host cells to which zoospores have attached, encysted **C** and penetrated the host via a penetration tube (PT). Bars: A = 5 μm, B = 10 μm

**Fig. 2:**
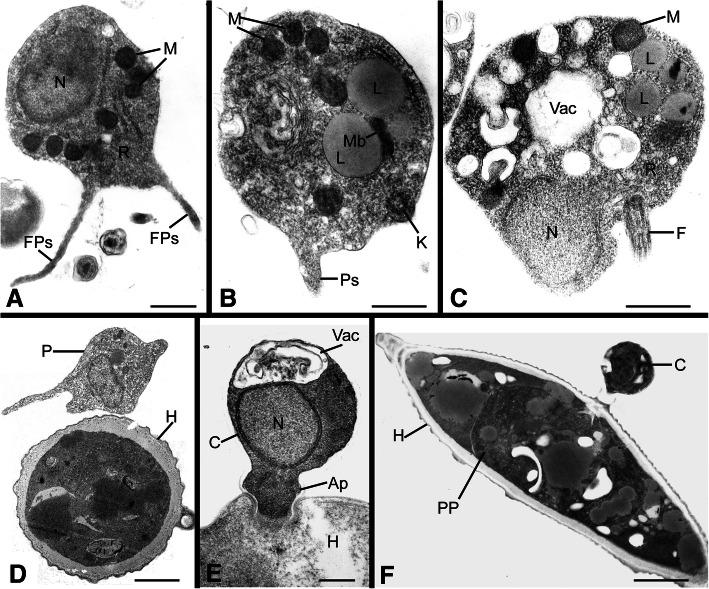
Transmission electron micrographs of life stages representative of *Aphelidiaceae*. **a** Motile zoospore with filose pseudopodia (FPs) and containing a nucleus (N), multiple mitochondria (M), and dispersed ribosomes (R). **b** Motile zoospore with a pseudopodium (Ps), multiple lipid globules (L) and an adjacent microbody (Mb), and a kinetosome (K). **c** Motile zoospore with a posterior flagellum (F) and central vacuole (Vac). **d** Amoeboid zoospore (P) approaching a host cell. **e** Encysted zoospore (C) containing an anterior vacuole (Vac) and a nucleus, attached to host via an appressorium (Ap). **f** An infected host cell, with a zoospore cyst attached and the parasitoid plasmodium (PP) inside the host. Figs. A, B, D, E, F* =Am. occidentale* (FD 01); Fig. C = *Aph. desmodesmi* strain FD 104. Bars: A–E = 5 μm, F = 2 μm

**Fig. 3:**
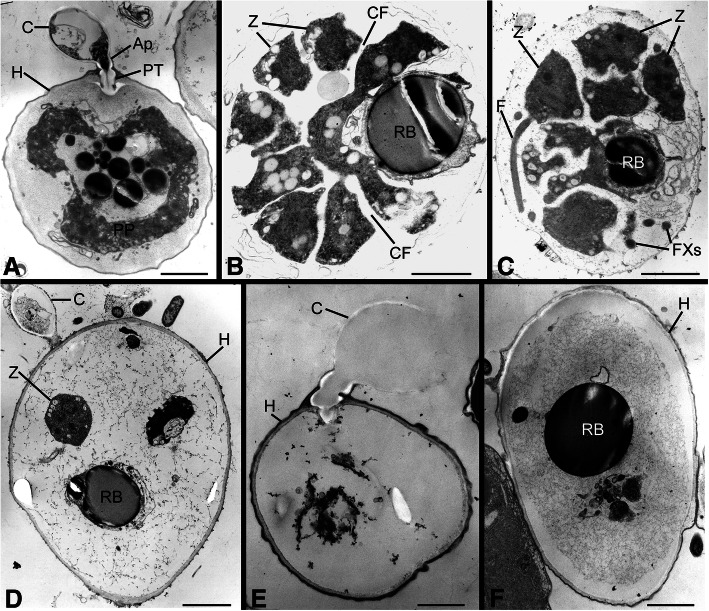
Transmission electron micrographs of life stages representative of *Aphelidiaceae*. **a** Host cell (H) with a remnant cyst (C) attached to host via an appressorium (Ap) and infecting the host with a penetration tube (PT). Parasitoid plasmodium (PP) has engulfed much of the host cytoplasm. **b** Parasitoid plasmodium cleaved into multiple uninucleate cells (Z) delineated by cleavage furrows (CF); a residual body (RB) remains. **c** Cleaved zoospores, one with a flagellum (F); flagellar cross-sections (FXs) also visible. **d** An evacuated sporangium that retained a zoospore and the residual body. **e** An empty sporangium with ephemeral remnant of zoospore cyst. **f** An evacuated sporangium with a residual body. Figs. A, E = *Am. occidentale* (FD 01); Fig. B–D = *Aph. desmodesmi* (FD 104); Fig. F = *Aph. protococcorum* (FD 95) Bars: A–D, F = 2 μm, E = 1 μm)

The aphelid life-cycle is similar among the included taxa. When viewed with light or transmission electron microscopy, the motile zoospore may be amoeboid (Figs. 1 and 2a, b), with one or more pseudopodia that may be either broad (e.g. “lamellipodium”, *Pa. tribonematis*, see Karpov et al. 2017a, Fig. 2c, d) or thin (e.g. “filopodia”, *Aph. desmodesmi*, see Letcher et al. 2017; “stiletto pseudopodium”, *Aph. chlorococcorum f. majus*, see Gromov 1976, Figs. 1 and 2). The zoospore may also be round or oval and without pseudopodia (Fig. 2c) (e.g. *Ps. drebesii,* see Schweikert and Schnepf 1996). The motile zoospore approaches the host and often contours its surface to that of the host (Fig. 2d), encysts on the host, attaches with an appressorium (Fig. 2e, Fig. 3a), and penetrates the host with a penetration tube (Fig. 1b, Fig. 3a). A posterior vacuole within the cyst (Fig. 2e) pushes the cyst contents into the host through the penetration tube. The endobiotic parasitoid becomes a phagotrophic amoeba. As the parasitoid grows it becomes a plasmodium that engulfs host cytoplasm (Fig. 2f, Fig. 3a), finally containing one or two residual bodies (Fig. 3b–d, f). At maturity the plasmodium is multinucleate, with a central vacuole and a residual excretion body. The plasmodium divides into numerous uni-nucleate cells (Fig. 3b, c), which are subsequently released from the empty host cell to further infect other host cells. An unreleased zoospore occasionally may remain in an evacuated sporangium (Fig. 3d), and the empty zoospore cyst may or may not persist (Fig. 3d, e). A resting spore may or may not be formed.

Variation in character states that may be taxonomically informative are: spore size and shape, flagellum length, and nature of pseudopodia; morphology of the zoospore cyst; size of residual body in the plasmodium; presence or absence of a resting spore; and shape and wall construction of the resting spore. Character states and hosts of taxa are summarized (Table 1).

**Table 1:** Morphological characters, character states, and hosts for aphelid taxa

Taxon	Spore size (μm), shape	Flagellum (L, μm)	Cyst	Resting spore size (μm), shape	Host
Aphelidium: Aph. chaetophorae	2.7–3, spherical	9	not observed	not observed	*Chaetophora elegans*
Aph. chlorococcorum f. chlorococcorum	1.5–2, “stiletto” pseudopodia	8	sessile or short stalk	7–13 × 5–6.5, ellipsoidal	Scenedesmus, chlorococcus algae (green algae)
Aph. chlorococcorum f. majus	2–3, spherical, conic “stiletto” anterior pseudopodium	14	stalked	5 × 8, ellipsoidal	Kirchneriella obesa, Ankistrodesmus (green algae)
Aph. deformans	2-3, spherical	unknown	not observed	12–30 diam, round to oval	*Coleochaete soluta* (green alga)
*Aph. desmodesmi*	~2, spherical, sub-spherical, angular numerous thin filopodia	~ 6	stalked	not observed	Desmodesmus opoliensis (green alga)
Aph. melosirae	4 × 6, pleomorphic	~ 10	sessile	12–14 × 10, oval	*Melosira varians* (diatom)
Aph. tribonematis	2–3, oval, numerous filopodia	6–8	sessile or short stalk	6–7, spherical, residual body outside	Tribonema gayanum, Botridiopsis intercedens (yellow-green algae)
Paraphelidium: Pa. letcheri	2–2.5, spherical, With a lamellipodium and subfilopodia	8–10	sessile or short stalk	6–8, spherical, single wall, residual body outside	Tribonema gayanum
Pa. tribonematis	2–2.5, oval, a broad anterior lamellipodium; a few lateral and anterior subfilopodia	~ 7	stalked	8–10, ellipsoid, two-walled, residual bodies between the two walls	*Tribonema gayanum*
*Amoeboaphelidium*: *Am. achnanthis*	~2 long	unknown	unknown	unknown	Achnanthes lanceolata (diatom)
Am. chlorellavorum	1–2, amoeboid	unknown	sessile	3–7, spherical	Chlorella spp. (green alga)
Am. occidentale	1.3–2.7, spherical, sub-spherical, elongate	7–10	stalked	unknown	Scenedesmus dimorphus
*Am. protococcorum*	2–4, spherical to elongate, numerous pseudopodia, thin trichipodia, thick lobopodia	~ 7	sessile	4–6 × 5–7, oval	Scenedesmus, Protococcus, chlorococcus algae
Am. radiatum	1–3, spherical, numerous filopodia	unknown	stalked	unknown	Kirchneriella, Ankistrodesmus, chlorococcus algae
Pseudaphelidium: Ps. drebesii	3 × 5, elongate	15	sessile	unknown	Thalassiosira punctigera (marine diatom)

Diversity within the group is indicated by 18S rRNA gene sequence molecular affinity of members of three genera, with numerous environmental sequences from diverse habitats (e.g., Karpov et al. 2017b). Molecular data for *Pseudaphelidium* are not available.

## TAXONOMY

Division: *Opisthokonta* Cavalier-Smith, *in* Rayner et al. (eds), *Evol. Bio. Fungi*: 339 (1987).

Kingdom: *Fungi* R.T. Moore, *Bot. Marina* 23: 371 (1980).

Phylum: *Aphelidiomycota* Tedersoo et al., *Fungal Diversity* **90**: 147 (2018); *Index Fung.* ID: 553990.

*Synonym*: *Aphelida* Karpov et al., *Frontiers Microbiol.* **5**: 112 (2014).

*Type*: *Aphelidium* Zopf 1885.

*Description*: “Opisthokont intracellular parasitoids of algae with phagotrophic amoeboid vegetative stage. Invasive cyst with short infective tube of penetration apparatus. Zoospores with pseudopodia and/or posteriorly directed functional or rudimentary flagellum” (Karpov et al. 2014a).

Subphylum: *Aphelidiomycotina* Tedersoo et al., *Fungal Diversity* **90**: 147 (2018); *Index Fung.* ID: 554031.

*Type*: *Aphelidium* Zopf 1885.

Class: *Aphelidiomycetes* Tedersoo et al., *Fungal Diversity* **90**: 147 (2018); *Index Fung.* ID553991.

*Synonym*: *Aphelidea* B.V. Gromov, *Zool. Zhurn.* **79**: 521 (2000).

*Type*: *Aphelidium* Zopf 1885.

*Description*: “Amoeboid endobiotic parasitoids of algae. Dispersal zoospores or amoebae attach to a new host cell and encyst, (either sessile on the substrate or producing a stalk; “apophyse”; Gromov 2000). Amoeboid body penetrates into the host’s cell through a cyst stalk. The intracellular amoeba engulfs the contents of the host’s cell, forming food vacuoles which transport food into the central digestive vacuole. An excretory body is formed in the digestive vacuole. The amoeboid trophont grows into a plasmodium, which totally replaces the cytoplasm of a host cell; the multinuclear plasmodium develops into an unwalled sporangium and divides into uninucleate amoeboid cells or flagellated zoospores. No specialized cell wall is formed by the parasitoid around the sporangium. Some species form intracellular resting spores” (Karpov et al. 2014a).

Order: *Aphelidiales* Tedersoo et al., *Fungal Diversity* **90**: 147 (2018); *Index Fung.* ID: 553992.

*Synonyms*: *Aphelidida* B.V. Gromov, *Zool. Zhurn.* **79**: 521 (2000).

*Aphelidida* Cavalier-Smith, Eur*. J. Protist.* **49**: 155 (2013); nom. Illegit. (Art. 54.1).

*Type*: *Aphelidium* Zopf 1885.

*Diagnosis*: As for the class.

Family: *Aphelidiaceae* Tedersoo et al., *Fungal Diversity* **90**: 147 (2018); *Index Fung.*ID: 553993.

*Synonym*: *Aphelididae* B.V. Gromov, *Zool. Zhurn.* **79**: 521 (2000).

*Type*: *Aphelidium* Zopf 1885.

*Diagnosis*: As for the class.

*Note*: The family comprises the genera: *Aphelidium*, *Paraphelidium*, *Amoeboaphelidium*, and *Pseudaphelidium*.

**Aphelidium** Zopf, *Morph. Biol. Pilzthiere*: **30** (1885).

*Type*: *Aphelidium deformans* Zopf, *Morph. Biol. Pilzthiere*: **30** (1885).

*Diagnosis*: “Parasitoid of various algae, forming round or oval zoospores with one posterior flagellum with an acroneme and one or several lipid grains. Vegetative development as described for the class. Resting spores round or oval, with a thick smooth cell wall. The excretory body is ejected from the spore into the space between the walls of the spore and the destroyed cell” (Gromov 2000).

**Aphelidium chaetophorae** Scherff., *Arch. Protistenk.* **52**: 47 (1925).

*Type*: Scherffel (*Arch. Protistenk.* **52**: taf. 3, figs. 113–122, 1925 – ***lectotype designated here***, MBT 384671).

*Diagnosis*: Scherffel (1925) observed neither formation of a zoospore cyst nor penetration of the parasite into the host cell. He did observe the parasite plasmodium within the host, with multiple digestive vacuoles. The parasite often caused hypertrophy of the infected cell. In the sporangium zoospores were initially spherical, ~ 2.7 μm diam, with a single flagellum ~ 9 μm long, and the zoospore may have possessed a posterior cavity and 2–3 contractile vacuoles. Prior to discharge zoospores became ovoid, 3–4 μm in length; zoospores were passively discharged, quiescent after exit, and then suddenly became motile, like many chytrids. Resting spores were not observed.

*Note:* Gromov (1976) wrote “The parasite develops in the same way as the other species described by Scherffel (1925)”. However, Gromov (2000) noted that the morphology of this species “does not correspond to the presented diagnosis of the genus”, without providing specifics. In our opinion, *Aph. chaetophorae* is not a doubtful species because *Aph. deformans* (the type species) is similar to *Aph. chaetophorae*, and in neither were encysted zoospores and empty cysts observed.

**Aphelidium chlorococcorum** Fott, *Univ. Carol. Biol.* **3** (2): 231 (1957); as ‘*chlorococcarum*’.

f. **chlorococcorum.**

*Type*: Fott (*Univ. Carol. Biol.* **3** (2): 237, figs 1–11, 1957 – ***lectotype designated here***, MBT 384672).

*Diagnosis*: Parasite of representatives of various genera of chlorococcus algae. “*Chlorococcus* is generally referred to algae in the order *Chlorococcales* (e.g. *Scenedesmus*, *Chodatella*), but Fott (1957) also included hosts from genera other than those in *Chlorococcales* e.g. *Oocystis*, *Actinastrum*, *Ankyra*). Zoospores 1.5–2.0 μm diam, flagellum about 8 μm long. Zoospore cyst sessile or with a short stalk. Resting spores ellipsoidal, 7.0–13.0 × 5.0–6.5 μm. Parasite’s ultrastructure from mass culture of *Scenedesmus armatus* was investigated by Schnepf and colleagues (Schnepf et al. 1971)” (Gromov 2000). Thallus morphology has been studied by Fott (1957) and the ultrastructure has been examined (Gromov 1976; Gromov and Mamkaeva 1975; Schnepf et al. 1971). Molecular sequence data are not available.

*Notes*: The epithet change (*chlorococcarum* --- > *chlorococcorum*) is the corrected Latin form for “of the *Chlorococcales*”.

Fott’s (1957) illustrations show oval zoospores with a single lipid globule and no pseudopodia (Fig. 3) and sessile zoospore cysts (Figs. 1 and 2). Schnepf et al. (1971) illustrated a zoospore cyst with a stalk (Figs. 2, 8) as well as flagellar cross sections with cleaved zoospores in the host cell. Gromov and Mamkaeva (1975) illustrated longitudinal sections through posteriorly uniflagellate zoospores (Figs. 1 and 2), the flagellum terminating with a thin acroneme; the zoospore also appears to have multiple (two or more) thin pseudopodia located at the anterior end of the zoospore. Gromov (1976) used the same two illustrations (Figs. 1 and 2), designated in the figure legend, however, as representing *Aph. chlorococcorum* f*. majus*. Gromov (2000) again repeated one of the illustrations (Fig. 1) as representing *Aph. chlorococcorum* f*. chlorococcorum*. Gromov (2000) suggested that “… the apically located stiletto-pseudopodium … seems to serve for attachment to an algal cell”.

**Aphelidium chlorococcorum** f. **majus** B.V. Gromov & Mamkaeva [as ‘*chlorococcarum*’], *Acta Protozool*. **7**: 266 (1970).

*Type:* Gromov & Mamkaeva (*Acta Protozool*. **7**: plate 1, figs 1–9, 1970 – ***lectotype designated here***, MBT 384673).

*Diagnosis*: “Zoospores 2.0–3.0 μm in diameter, flagellum about 14 μm long” (flagellum proper 7–9 μm, acroneme~ 5 μm long) (Gromov 2000; Gromov and Mamkaeva 1970b). Thallus morphology has been studied (Gromov and Mamkaeva 1970b), and the ultrastructure of zoospores and vegetative structures investigated (Gromov and Mamkaeva 1975). Molecular sequence data are not available.

*Note*: The *forma* designation ‘*majus’* refers to the larger dimensions of the zoospore and its flagellum when compared with that of *Aph. chlorococcorum* f. *chlorococcorum*.

**Aphelidium deformans** Zopf, *Morph. Biol. Pilzthiere*: 30 (1885).

*Type*: Zopf (*Morph. Biol. Pilzthiere*: **30**: taf. IV, figs. 1–17, 1885; ***lectotype designated here***, MBT 384674).

*Diagnosis*: “Parasitoid of the green alga *Coleochaete*. The infected cell is deformed, becoming abnormally large; the cell wall is thickened. Zoospores 2–3 μm in diameter. Zoospore cyst not observed. Resting spore round to oval, 12–30 μm in diameter, with a large lipid grain” (Gromov 2000). Thallus morphology has been illustrated (Zopf 1885) but the ultrastructure has not been examined, and molecular sequence data are not available.

*Note*: Zopf's illustrations (Zopf 1885, plate 4, Figs. 1-17) show spores within host cells (his figures 5 and 6), but do not show spore flagellation, spore release, or spore encystment upon the host.

**Aphelidium desmodesmi** Letcher, *J. Eukar. Microbiol.* **64**: 658 (2017).

*Type*: Letcher et al. (*J. Eukar. Microbiol.* **64**: 659, fig. 2, 2017 – holotype).

*Diagnosis*: “Endobiotic parasitoid of *Desmodesmus armatus* (Chodat) E. Hegewald, as an intracellular phagotrophic amoeba that engulfs the host cytoplasm, develops into an endobiotic plasmodium, becomes multinucleate, and cleaves into zoospores. Zoospores 1.6–1.9 μm diameter, with a posterior whiplash flagellum 7–9 μm in length (flagellum proper + acroneme), and multiple filose pseudopodia radiating from the zoospore body; zoospores contain a nucleus, a microbody-lipid globule complex (MLC) with multiple lipid globules 0.3–0.4 μm diameter and multiple spherical mitochondria 0.2–0.3 μm diameter with flat and rhomboid cristae, a Golgi apparatus composed of stacked cisternae anterior to the kinetosome, a non-flagellated centriole parallel or at a slight angle to the kinetosome, and dispersed ribosomes. Zoospore cysts stalked, 1.8–2.1 μm diameter. Resting spores not observed” (Letcher et al. 2017). Thallus morphology and ultrastructure have been studied (Letcher et al. 2017). GenBank accession: KY249641 (SSU-ITS1–5.8S-ITS2-LSU rDNA).

**Aphelidium melosirae** Scherff., *Arch. Protistenk.* **52**: 39 (1925).

*Type*: Scherffel (*Arch. Protistenk.* **52**: taf.2, figs. 87–90; taf. 3, figs. 91–101, 1925 – ***lectotype designated here***, MBT 384675).

*Diagnosis*: “Parasitoid of the diatom alga *Melosira varians* Ag. Zoospores pleomorphic, 4 × 6 μm, with several refractive grains. Flagellum about 10 μm long. When leaving host’s cell and during some time after, zoospores are amoeboid and move like amoebae. Zoospore cyst sessile. Resting spore 12–14 × 10 μm in size” (Gromov 2000). Thallus morphology has been studied (Scherffel 1925), but not the ultrastructure, and molecular sequence data are not available.

*Notes*: Scherffel’s illustrations (Scherffel 1925, plate 2, figs. 87–90; plate 3, figs. 91–101) show pleomorphic zoospores: round (fig. 98a), oval (fig. 98b), and irregular (fig. 98c– d), with one or more lipid globules, and zoospores with apically located pseudopodia (fig. 98d); a sessile zoospore cyst (figs. 87–89) is also illustrated.

Karpov et al. (2014b) studied the molecular phylogeny and ultrastructure of a strain identified as *Aphelidium* aff. *Melosirae* (strain P-1 CALU, GenBank KJ566931) infecting the host *Tribonema gayanum*. *Aphelidium* aff*. Melosirae* is considered similar to (has an affinity with) *Aph. melosirae*, whose host is *Melosira varians*, because, of the six species known for the genus, strain P-1 appears to be morphologically most similar to *Aph. melosirae*. *Aphelidium* aff*. Melosirae* is therefore an undescribed strain, and to decide whether strain P-1 belongs to *Aph. melosirae* or not, the morphology and molecular phylogeny of *Aph. melosirae* parasitizing *Melosira varians* need to be studied (Karpov et al. 2014b).

**Aphelidium tribonematis** Scherff., *Arch. Protistenk.* **52**: 44 (1925); as ‘*tribonemae*’.

*Type*: Scherffel (*Arch. Protistenk.* **52**: taf. 3, figs. 102–112, 1925 – ***lectotype designated here***, MBT 384676).

*Diagnosis*: “Parasitoid of a yellow-green alga, *Tribonema*. Zoospores 2–3 μm in diameter, flagellum about 6–8 μm long with acroneme about 5 μm long. Zoospores amoeboid, capable of forming numerous thin pseudopodia. Zoospore cyst sessile or with a short stalk. The development of *A. tribonemae* has been observed in *Tribonema gayanum* Pasch. and *Botridiopsis intercedens* Visch. & Pasch.” (Gromov 2000). Thallus morphology has been studied (Gromov 1972; Scherffel 1925; Karpov et al. 2016). Thallus ultrastructure has not been studied. GenBank accession: KY129663 (partial SSU rDNA; Karpov et al. 2016).

*Note*: Scherffel’s illustrations (Scherffel 1925, plate 3, figs. 102–112) show oval zoospores; the zoospore cyst appears as either very short-stalked (fig. 102) or sessile (figs. 102–103, 105).

Karpov et al. (2016) studied morphology and molecular phylogeny of strain X-102, identified as *Aph. tribonematis*. The zoospores of strain X-102 can produce a lamellipodium and filopodia from different sides of the cell body; the zoospore cyst of strain X-102 has a short stalk (Fig. 2i, k).

### Doubtful species of *Aphelidium*

*Aphelidium lacerans* Bruyne, *Arch. Biol. (Paris)* **10**: 74 (1890).

The morphology of this species “does not correspond to the presented diagnosis of the genus” (Gromov 2000). de Bruyne (1890) illustrated anteriorly uniflagellate (“un cil implanté a la partie antérieure”) zoospores for this organism, and the zoospores contained grains of chlorophyll (“zoospores renfermant de la Chlorophylle encore intacte”; figs. 28–29). The anterior cilium would exclude it from the Opisthokonts. While describing this organism as the new species *Aphelidium lacerans* (de Bruyne 1890: 74), he also figured it as “*Olpidium lacerans*” (de Bruyne 1890: 104, figs. 28–32). Sparrow (1960) rejected *Aph. lacerans*, stating: “Not a fungus. The zoospores contain chlorophyll residue. The figure refers to the monad *Aphelidium lacerans*”, but Sparrow did not question its placement in *Aphelidium*. Dangeard (1890), in a brief description of putatively the same taxon, illustrated uniflagellate, amoeboid zoospores (figs. 22–23), but because he did not observe germination, he could not establish its relationship with *Aphelidium*.

**Paraphelidium** Karpov et al., *J. Eukar. Microbiol.* **64**: 211 (2017).

*Type*: *Paraphelidium tribonematis* Karpov et al. 2017a.

*Diagnosis*: Zoospores swim with a posteriorly oriented flagellum or move like amoebae with an immobile flagellum. Amoeboid zoospore can produce a short, broad anterior lamellipodium with subfilopodia from the lamellipodium and separate filopodia. Mature resting spore (sporocyst) is ellipsoid and covered with one or two walls. The two-walled morphology of the resting spore is present only in the type species (Karpov et al. 2017a), the resting spore of the second described species *P. letcheri* having only a single wall (Karpov et al. 2017b).

**Paraphelidium letcheri** Karpov & Torruella, *J. Eukar. Microbiol.* **64**: 575 (2017).

*Type*: Karpov et al. (*J. Eukar. Microbiol.* **64**: 576, fig. 2, 2017 – holotype; CCPP ZIN RAS collection X–129 – ex-type culture).

*Diagnosis*: “Crawling flagellated zoospores have a body up to 4 μm long and able to produce a lamellipodium with subfilopodia up to 1.8 μm in length; swimming zoospores with spherical body 2–2.5 μm in diameter, and a flagellum 8–10 μm including an acroneme of 4 μm. Large residual body associated with one or two lipid globules totally occupies a central vacuole of plasmodium. Sporocyst spherical 6–8 μm in diameter with smooth wall” (Karpov et al. 2017b). Parasitoid of *Tribonema gayanum*. Thallus morphology has been studied (Karpov et al. 2017b), but not the ultrastructure. GenBank accession: KY412789 (partial SSU rDNA).

*Note*: *Paraphelidium letcheri* is distinguishable from the type species, *P. tribonematis,* by a much larger residual body associated with big colorless lipid globules in the plasmodium, and by the single-walled resting spore.

**Paraphelidium tribonematis** Karpov et al., *J. Eukar. Microbiol.* **64**: 211 (2017); as “*tribonemae*”.

*Type*: Karpov et al. (*J. Eukar. Microbiol.* **64**: 207, fig. 2a–e, 2017 – holotype; CCPP ZIN RAS collection X–108 – ex-type culture).

*Diagnosis*: “Zoospores with body length of 2–2.5 μm, with a broad anterior lamellipodium and a few anterior and lateral subfilopodia, flagellum 7 μm in length with variable length of acroneme (1–3.5 μm). Mature resting spore (sporocyst) is ellipsoid and covered with two walls” (Karpov et al. 2017a). Parasitoid of *Tribonema gayanum*. Thallus morphology and ultrastructure have been studied (Karpov et al. 2017a). GenBank accession: KX576680 (partial SSU rDNA).

**Amoeboaphelidium** Scherff., *Arch. Protistenk.* **52**: 52 (1925).

*Diagnosis*: “Parasitoids of various species of algae. Amoeboid zoospores with or without posterior pseudocilium, forming flat hyaline pseudopodium with subfilopodia, or filopodia of different length. Vegetative development as described for the class. Resting spores rounded to oval, with a thick cell wall” (Karpov et al. 2014a).

*Type*: *Amoeboaphelidium achnanthis* Scherff. 1925.

*Note*: *Amoeboaphelidium* was originally characterized as having non-flagellate, pseudopodiate amoeboid zoospores (“Schwärmer ohne Geißel”: “swarmers [spores] without [a] cilium”), the distinction that differentiated it from the flagellated *Aphelidium* (“Schwärmer mit einer, nachschleppenden Geißel”: “swarmers [spores] with a trailing cilium”) (Scherffel 1925). As the main feature of opisthokonts is a posteriorly uniflagellate zoospore, Karpov et al. (2013) reinvestigated the ultrastructure of the amoeboid *Amoeboapheldium protococcorum* and found a *pseudocilium* that was not described earlier (Gromov and Mamkaeva 1970a). The pseudocilium is a “permanently immotile posterior projection containing microtubules, and so it may be considered a reduced flagellum. Thus, the dispersal stage of the life-cycle in all known aphelids is a true opisthokont zoospore” (Karpov et al. 2013). This observation is correct for *A. protococcorum* (Karpov et al. 2013; Letcher et al. 2015) and *A. occidentale* (Letcher et al. 2013, 2015), but can only be presumed for the other species of *Amoeboaphelidium* that have not been examined ultrastructurally.

**Amoeboaphelidium achnanthis** Scherff. *Arch. Protistenk.* **52**: 52 (1925); as “*achnanthidis”*.

*Type*: Scherffel (*Arch. Protistenk.* **52**: 52, 1925 – holotype).

*Diagnosis*: Thallus morphology is descriptive only, as Scherffel (1925) did not illustrate this taxon. “Parasitoid of the diatom alga *Achnanthes*, amoebae about 2 μm long” (Gromov 2000). Thallus ultrastructure has not been studied, and molecular sequence data are not available.

**Amoeboaphelidium chlorellavorum** B.V. Gromov & Mamkaeva, *Acta Protozool.* **6**: 224 (1968).

*Type*: Gromov & Mamkaeva (*Acta Protozool.* 6: pl. 1, figs. 9, 14–15, 1968 – ***lectotype designated here***, MBT 384677; CALU x-2 – ex-type culture).

*Diagnosis*: “Parasitoid of some species of *Chlorella*. Amoeba about 1 μm diameter, extracellular cysts without a discernable stalk” (Karpov et al. 2014a). Thallus morphology (Gromov and Mamkaeva 1968) and thallus ultrastructure (Gromov 1976; Gromov and Mamkaeva 1970c) have been studied. Molecular sequence data are not available.

*Note*: Gromov and Mamkaeva (1968) distinguished this taxon from *Amoeboaphelidium protococcarum* primarily on the basis of host specificity and shape of the dormant (resting) spores. Gromov and Mamkaeva (1970c) illustrated a sessile zoospore cyst (plate 1), and Gromov (2000) stated that the extracellular zoospore cyst was without a discernable stalk.

**Amoeboaphelidium occidentale** Letcher, *Mycologia* **107**: 528 (2015).

*Type*: Letcher et al. (PLoS ONE 8: e56232, doi:10.1371/journal.pone.0056232, fig. 6B, 2013 –holotype; FD01, Sapphire Energy FD01 – ex-type culture).

*Diagnosis*: “Amoeboid endobiotic parasitoid of *Scenedesmus dimorphus*. Amoeboid zoospores 1.7–2.5 μm diameter, with a posterior pseudocilium and multiple anterior and lateral filose pseudopodia; zoospores contain a nucleus, a microbody lipid-globule complex (MLC) with multiple lipid globules and multiple spherical mitochondria 0.25–0.5 μm diameter with lamellar cristae, endoplasmic reticulum backing the lipids in the MLC, and dispersed ribosomes. Zoospore cysts 1.3–2 μm diameter” (Letcher et al. 2015). Thallus morphology and ultrastructure have been studied (Letcher et al. 2013, 2015). GenBank accession: JX967274 (SSU-ITS1–5.8S-ITS2-LSU rDNA).

**Amoeboaphelidium protococcorum** B.V. Gromov & Mamkaeva, *Acta Protozool*. **6**: 224 (1968); as “*protococcarum*”.

*Type*: Gromov & Mamkaeva (*Acta Protozool*. **6**: pl. 1, figs. 1–8, 1968 – ***lectotype designated here***, MBT 384678; CALU x–1, ATCC 50289 – ex-type cultures).

*Diagnosis*: “Parasitoid of *Scenedesmus*, *Protococcus* and some other genera of protococcus algae; strains differ by the possible hosts (Gromov and Mamkaeva 1966, 1969b; Mamkaeva and Gromov 1969) and environmental conditions (Gromov and Titova 1973). Amoebae 2.0–4.0 μm in diameter with posterior pseudocilium 7 μm long. Resting spores oval, 4–6 × 5–7 μm” (Karpov et al. 2014a). Thallus morphology (Gromov and Mamkaeva 1966, 1968) and thallus ultrastructure (Gromov 1976; Gromov and Mamkaeva 1970a; Karpov et al. 2013; Letcher et al. 2015) have been studied. Variation among molecular karyotypes indicates possible taxon distinctions with this organism (Pinevich et al. 1997). GenBank accession JX507298 (SSU-ITS1–5.8S-ITS2-LSU rDNA).

*Notes*: The epithet change from “*protococcarum*” to “*protococcorum*” is the corrected Latinized form for “of the protococci”.

Letcher et al. (2015) and Ding et al. (2017) examined additional strains (FD 95, Sapphire Energy, Inc. and WZ01, respectively) of this taxon.

The amoebae form numerous pseudopodia, thin trichipodia (hair-like), and thick lobopodia. The amoeba cyst is attached to the host by a short stalk (e.g. Letcher et al. 2015: Fig. 3d–, e). Gromov and Mamkaeva (1969c) stated the “diameter of amoeba with retracted pseudopodia ~2–4 μm; parasite grows well on surface of solid media; contrast with *Amoeboaphelidium radiatum*, which grows only in semi-solid or liquid media; contrast with *Aphelidium chlorococcorum* that grows only in algae in liquid medium”.

**Amoeboaphelidium radiatum** B.V. Gromov & Mamkaeva, *Biologiia* **9**: 144 (1969).

*Type*: Gromov & Mamkaeva (*Biologiia* **9**: 142, figs. 1–8, 1969 – ***lectotype designated here***, MBT 384679; CALU x-3 – ex-type culture).

*Diagnosis*: “Parasitoid of the chlorococcus algae *Kirchneriella* and *Ankistrodesmus*. Amoebae 1–3 μm in diameter with limited motility, have very thin and long filopodia (10–12 μm). Development of the surface of solid culture media not observed” (Karpov et al. 2014a). Thallus morphology has been studied (Gromov and Mamkaeva 1969a), but ultrastructure details and molecular sequence data are not available.

*Note*: Gromov and Mamkaeva (1969a) stated that the motility of amoeboid spores was limited. Gromov and Mamkaeva (1969c) reported that the organism grew only in semi-solid or liquid media.

**Pseudaphelidium** Schweikert & Schnepf, *Arch. Protistenk.* **147**: 16 (1996).

*Diagnosis*: “Parasitoids of diatoms. Zoospores colourless, lacking conspicuous refractive granules, with a single opisthokont flagellum. A zoospore attaches to a host cell, encysts, penetrates into the cell interior, and develops into a phagocytotic plasmodium which ingests portions of host cytoplasm and includes them in a single big digestion vacuole. At the end of the trophic phase the plasmodium cleaves to form uni-nucleate amoeboid cells which encyst and give rise to new zoospores” (Schweikert and Schnepf 1996).

*Type*: *Pseudaphelidium drebesii* Schweikert amp; Schnepf 1996.

**Pseudaphelidium drebesii** Schweikert & Schnepf, *Arch. Protistenk.* **147**: 16 (1996).

Type: Schweikert & Schnepf (*Arch. Protistenk.* **147**: 13–15, figs. 1–16, 1996 – holotype).

*Diagnosis*: “Structure and development as described for the genus. Zoospores 5 μm long and 3 μm wide, flagellum 15 μm long. At the end of its development, the plasmodium consists of a thin hollow sphere. It cleaves to form globular cells from which the amoeboid cells arise. They are not very motile. They form cysts measuring 4–6 μm in diameter, which release 1 or 2 but generally 4 zoospores” (Schweikert and Schnepf 1996). Parasitoid of marine planktonic diatoms *Thalassiosira punctigera*. Thallus morphology and life-cycle have been studied (Schweikert and Schnepf 1996), and ultrastructural morphology has also been investigated (Schweikert and Schnepf 1997). Molecular sequence data are not available.

*Note*: This is the only described species in the genus.

### Key to the species of *Aphelidiaceae*

**Table:** 

1	Intracellular plasmodium cleaves, producing amoeboid cells that encyst, that then become posteriorly uniflagellate zoospores	Pseudaphelidium drebesii
	Intracellular plasmodium cleaves, producing aciliate aplanospores, or posteriorly immotile pseudociliate or motile ciliate (flagellate) zoospores	2
2 (1)	Cleavage product a zoospore with motile posterior flagellum	3
	Cleavage product aciliate (amoeboid) or with permanently immotile pseudocilium	8
3 (2)	Host: *Coleochaete* (green alga)	4
	Host: Not *Coleochaete*	5
4 (3)	Zoospore spherical, 2-3 μm diam; host. *C. soluta*	Aphelidium deformans
	Zoospore spherical to oval, 2.7 μm diam; host *C. elegans*	Aphelidium chaetophorae
5 (3)	Host: *Melosira* (diatom)	Aphelidium melosirae
	Host: *Scenedesmus*, chlorococcoid algae (green algae)	Aphelidium chlorococcorum f. chlorococcorum
	Host: *Kirchneriella*, *Ankistrodesmus* (green algae)	Aphelidium chlorococcorum f*.* majus
	Host: *Desmodesmus* (green alga)	Aphelidium desmodesmi
	Host: *Tribonema* (yellow-green alga)	6
6 (5)	Zoospore cyst sessile or with a short stalk; zoospores with a lamellipodium and filopodia from different sides of the zoospore body	Aphelidium tribonematis
	Zoospore cyst sessile; zoospores with a lamellipodium and filopodia from the lamellopodium	7
7 (6)	Resting spore 1-walled; residual body outside the wall	Paraphelidium letcheri
	Resting spore 2-walled; residual body between the two walls	Paraphelidium tribonematis
8 (2)	Cleavage product aciliate	9
	Cleavage product with permanently immotile pseudocilium; strains morphologically similar, genetically distinct	10
9 (8)	Host *Achnanthes* (diatom)	Amoeboaphelidium achnanthis
	Host *Chlorella* (green alga)	Amoeboaphelidium chlorellavorum
	Host *Kirchneriella*, *Ankistrodesmus* (green algae)	Amoeboaphelidium radiatum
10 (8)	GenBank JX507298	Amoeboaphelidium protococcorum
	GenBank JX967274	Amoeboaphelidium occidentale

## Abbreviations

Ap: Appressorium
C: Cyst
CF: Cleavage furrow
F: Flagellum
FPs: Filose pseudopodium
FXs: Flagellar cross section
H: Host
K: Kinetosome
L: Lipid globule
M: Mitochondrion
Mb: Microbody
N: Nucleus
P: Parasite
PP: Parasite plasmodium
Ps: Pseudopodium
PT: Penetration tube
R: Ribosomes
RB: Residual body
Vac: Vacuole
Z: Zoospore
